# A hidden HIV epidemic among women in Vietnam

**DOI:** 10.1186/1471-2458-8-37

**Published:** 2008-01-28

**Authors:** Thu Anh Nguyen, Pauline Oosterhoff, Anita Hardon, Hien Nguyen Tran, Roel A Coutinho, Pamela Wright

**Affiliations:** 1 Faculty of Public Health, Hanoi Medical University, Hanoi, Vietnam; 2 Medical Committee Netherlands Vietnam, Hanoi, Vietnam; 3 Amsterdam School of Social Science Research, University of Amsterdam, Amsterdam, The Netherlands; 4 National Institute of Hygiene and Epidemiology Hanoi, Vietnam; 5 Center for Infectious Disease Control, National Institute for Public Health and the Environment, Bilthoven, The Netherlands; 6 Academic Medical Center/University of Amsterdam, Department of Internal Medicine, The Netherlands

## Abstract

**Background:**

The HIV epidemic in Vietnam is still concentrated among high risk populations, including IDU and FSW. The response of the government has focused on the recognized high risk populations, mainly young male drug users. This concentration on one high risk population may leave other populations under-protected or unprepared for the risk and the consequences of HIV infection. In particular, attention to women's risks of exposure and needs for care may not receive sufficient attention as long as the perception persists that the epidemic is predominantly among young males. Without more knowledge of the epidemic among women, policy makers and planners cannot ensure that programs will also serve women's needs.

**Methods:**

More than 300 documents appearing in the period 1990 to 2005 were gathered and reviewed to build an understanding of HIV infection and related risk behaviors among women and of the changes over time that may suggest needed policy changes.

**Results:**

It appears that the risk of HIV transmission among women in Vietnam has been underestimated; the reported data may represent as little as 16% of the real number. Although modeling predicted that there would be 98,500 cases of HIV-infected women in 2005, only 15,633 were accounted for in reports from the health system. That could mean that in 2005, up to 83,000 women infected with HIV have not been detected by the health care system, for a number of possible reasons. For both detection and prevention, these women can be divided into sub-groups with different risk characteristics. They can be infected by sharing needles and syringes with IDU partners, or by having unsafe sex with clients, husbands or lovers. However, most new infections among women can be traced to sexual relations with young male injecting drug users engaged in extramarital sex. Each of these groups may need different interventions to increase the detection rate and thus ensure that the women receive the care they need.

**Conclusion:**

Women in Vietnam are increasingly at risk of HIV transmission but that risk is under-reported and under-recognized. The reasons are that women are not getting tested, are not aware of risks, do not protect themselves and are not being protected by men. Based on this information, policy-makers and planners can develop better prevention and care programs that not only address women's needs but also reduce further spread of the infection among the general population.

## Background

The HIV epidemic takes different forms in different countries, affecting selected sub-groups in the population. The spread may be mainly through heterosexual transmission as in Southern Africa or by injecting drug use as in China. Each country has to develop strategies and programs appropriate for its own epidemic, based on the most complete information available about risk groups and targets for interventions in both prevention and care.

The HIV epidemic in Vietnam is still classified as the concentrated stage with high prevalence among high risk populations, mainly injecting drugs users (IDU), and a low prevalence in the general population. The first case of HIV infection was reported in December 1990 in Ho Chi Minh City. By 1992, only 11 cases had been reported, but in 1993 there was a sharp increase and twelve years later, in December 2005, the cumulative number of reported HIV-infected people from the 64 provinces had grown to 104,111. Of these, 13,731 were new infections, 17,289 were AIDS patients and 10,071 had died. Estimates put the actual number of infections much higher. The HIV epidemic in Vietnam is predominantly drug-related; IDU have accounted for most (53%) of the recorded infections, although this data from surveillance may be incomplete. HIV prevalence in that group is still increasing, in 2005 at approximately 30%. The epidemic affects mainly young males; 64% of reported cases are men under 29 years of age [1].

In order to push back a potential generalized epidemic, the Vietnamese government has focused on the recognized high risk populations, mainly young male drug users. Among the nine objectives of the Vietnamese HIV/AIDS strategic plan 2006–2010, harm reduction accounts for nearly 20% of the total funds available, while PMTCT is allocated approximately 8% and the program to prevent sexually transmitted infections is planned to receive only 5% [2]. Among 58 international organizations working on HIV/AIDS prevention, 32 of them focus on drug users and nine on commercial sex workers and men who have sex with men; most of the others support programs aimed at raising awareness in general or on activities such as sentinel surveillance [3].

This concentration on one high risk population may leave other populations under-protected or unprepared for the risks and the consequences of HIV infection. In particular, women will not receive sufficient attention as long as the perception persists that the epidemic is among young males. But many women are already infected with HIV. In 2005, women were estimated to make up 33% of the 263,000 people thought to be living with HIV/AIDS nationwide [4]. Availability and accessibility of HIV care and support remain very low, but HIV-infected women may have even less access compared to other groups. The reason for that is partly because the infected women have been less visible to the policy-makers and planners than are the male IDU. To develop appropriate intervention strategies for women, a clearer picture is needed of their place in the HIV epidemic in Vietnam.

This study was carried out to explore the HIV infection situation among different social groups of women, as well as the behaviors that put them at risk for HIV infection, by collecting and systematically reviewing the information from available studies. Several agencies have conducted studies in Vietnam, often as preparation for or evaluation of HIV/AIDS programs, but the results are not always published in scientific journals. We reviewed evidence on HIV prevalence and determinants from both published and unpublished sources. The results will help to make clear to policy-makers and planners that there are enough HIV-infected women to merit more attention to their risks and needs. They will also suggest directions for possible interventions to reduce their risk and contribute to reduction of further spread of HIV among the general population.

## Methods

More than 300 documents in both English and Vietnamese (Table 1), including journal articles, survey reports, program reports, and evaluation studies, appearing in the period 1990 to end of 2005 were collected from various sources such as organizations and institutions working in HIV/AIDS in Vietnam, local and international conferences and meetings, libraries, online journals, and web pages of international organizations. The references from these studies were also reviewed to make the plan for data collection. Data collection lasted for two years (2004 – 2005).

**Table 1:** General information of reviewed papers*

Type of paper	In English	In Vietnamese
Quantitative research	105	35
Qualitative research	37	13
Report	90	30
Total	232	78

* The full list of reviewed papers and reports is available from the first author on request

The studies included here were first assessed according to the inclusion criteria shown in Table 2 and using a spreadsheet for data extraction. For the quantitative studies, reports using a sampling method suitable for obtaining representative results for the target populations were included; we also considered studies with larger sample sizes to be more likely to provide representative data. That meant excluding a number of available studies (34) with small and unrepresentative study samples.

**Table 2:** Inclusion criteria for quantitative studies

Aspect	Criteria
Sampling method	- Methods must be clearly described
	- Good method of data collection:
	∘ High risk group: Sampling frame should be made prior the survey.
	∘ Low risk group: household survey was weighted more than facility-based survey
Sample size	Representative (based on sample size calculation and estimated size of relevant population in a specific province).
	- High risk group: sample included at least 20% of high risk population identified in mapping stage
	- Low risk group: sample included at least 200 people/province
Interview method	Face-to-face and computer based interviews were weighted more than self-administered interviews
Response rate	> 95%
Frequency of data type	If a study was the only one reporting on key data, it was considered to be included

The reports were then analyzed using a spreadsheet for data extraction that included the methods and their constraints, study groups, behavioral and epidemiological data, study period and location, potential biases, and comments on quality of papers. Data from different sources were compared to triangulate and adjust information. Study design, sampling method, sample size, response rate and study instruments were reviewed critically to provide a deeper understanding of the data presented in each paper.

Triangulation of reviewed data was conducted between and within quantitative and qualitative papers. For example, data on sexually transmitted infection (STI) and HIV prevalence and on condom use were cross-checked to evaluate the data consistency (and probably validity), as were condom use with female sex workers (FSW) as reported by IDU and condom use with IDU as reported by FSW.

The study also made use of the most recent report on HIV/AIDS Estimates and Projections 2005–2010, prepared and published by the Ministry of Health of Vietnam, to compare the estimates and projected numbers of HIV infection with the reported numbers. This estimation and projection process for the period 2005 – 2010 used the same data and assumptions to describe the development of the epidemic since 1991. Sentinel surveillance data from 1994 to 2002 were carefully reviewed and triangulated with special surveys such as behavior surveillance surveys and household surveys, and discussed with local staff working on data collection. Trends of HIV prevalence were described for injecting drug users, sex workers, STI clinic attendees, antenatal clinic attendees and military recruits. The population sizes for IDU, FSW and the general male and female populations were taken from reports of the Ministry of Labor, Invalids and Social Affairs, the Provincial AIDS Committees and the General Statistics Office. The HIV prevalence in each population and the population sizes were entered into EPP (Estimation and Projection Package) and SPECTRUM software to estimate and project the numbers of HIV infections and AIDS cases in Vietnam [4]. This software were developed by UNAIDS; it was introduced to the Ministry of Health by the World Health Organization, because it had been successfully applied other countries with heterosexual epidemics. In countries with concentrated epidemics, this software is used to make national estimates based on prevalence in high risk populations and estimations of the size of the high risk populations. The Spectrum software helps to obtain estimates of mortality, numbers of people in need of certain interventions, and population projections [5].

In the following analysis, the data from the above-mentioned sources were used to draw conclusions about the probable numbers of women and the probable contributions of different risk behaviors in the HIV epidemic among women in Vietnam.

## Results

### Epidemiology of HIV in women – their place in the epidemic

The first case of a woman infected with HIV was detected in 1990; by 2005 the reported cumulative number of HIV-infected women was 15,633. According to reports from the Ministry of Health in 2005, the male-to-female ratio had remained stable at around 6:1 since 1993 [2]. Estimations and projections about the development of the epidemic in the early years did not disaggregate the data for sex, so that we could not compare the early expectations with the actual development of the epidemic among women.

Comparing the estimations for the year 2005 [4] to the reports for that year, the number of infected women reported (15,633) accounted for only 16% of the estimated number (98,500), which suggests that about 83,000 women infected with HIV went undetected. In 1991, the estimated cumulative number of HIV-infected women was only 35 in the whole country. By the same estimation process, fourteen years later (2005) the cumulative number of HIV-infected women would have reached 98,500, of whom 16,000 would by then be AIDS cases, and 12,000 would have died of AIDS.

**Figure 1:**
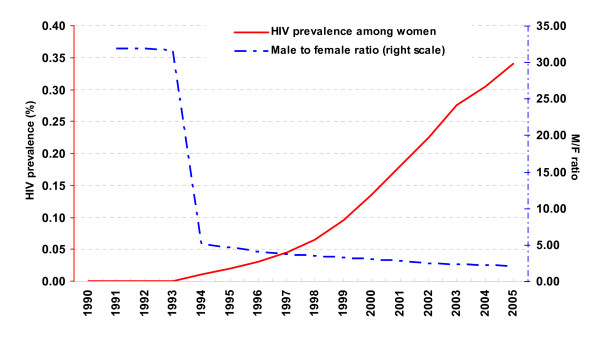
Projection from 2002 of trends in HIV prevalence among women and in male-to-female ratio of HIV infections in Vietnam (medium scenario), showing estimations based mainly on sentinel surveillance data. Source: HIV/AIDS estimation and projection 2005 – 2010. Ministry of Health.

It was predicted not only that the number of women infected with HIV would increase but also that the male-to-female ratio would decrease, from the estimated 5.1 in 1994 to only 2.03 in 2005 (Figure 1). The estimated HIV prevalence among women of reproductive age was expected to reach 0.34% in 2005, again according to these estimates [4]. Special household surveys made in Ho Chi Minh City, Thaibinh, and Haiphong in 2005 produced similar figures, showing HIV prevalence among women in general to be 0.6%, 0.1%, and 0.2%, respectively [6]. Particularly, the survey in Haiphong revealed an HIV prevalence among young women aged 25–29 of 1.1%, which would be indicative of a generalized epidemic [7].

HIV prevalence studies confirmed the alarming increase in HIV infection rates in identified high-risk populations, as well as transmission from them to the general population. Most of the data comes from sentinel surveillance sites where HIV infection rates in pregnant women are monitored. The first HIV-infected pregnant women in Vietnam were identified in 1993. The HIV prevalence among pregnant women then increased from 0.03% in 1994 to 0.37% in 2005. However, the latest sentinel surveillance in 2005 showed that HIV prevalence among this population in Thainguyen, Hanoi, and Quangninh had reached 2.0%, 1.25%, and 1.0%, respectively [2]. Of the 1.8 – 2 million women who give birth annually, an estimated 3,000 HIV positive women delivered in 2000 [8], 6000 in 2002 [8], and 7,000 – 8,000 in 2005 [1].

But the number of infected men in 2005 as reported by the Ministry of Health, 88,478, was 40% of the expected 220,900, suggesting a detection rate for men more than double that for women (16%) [4].

### Subgroups of women and HIV infection

**Figure 2:**
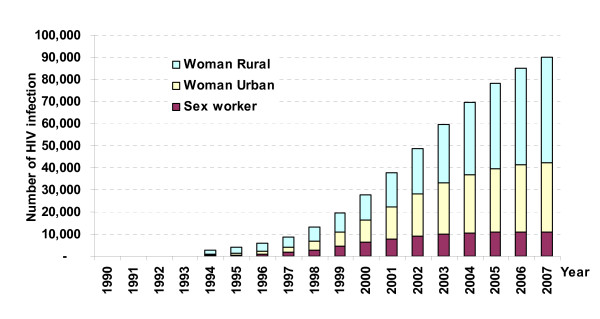
Projection from 2002 of trend in HIV infections among different sub-groups of women in Vietnam (medium scenario) showing estimations based mainly on sentinel surveillance data. Source: HIV/AIDS estimation and projection 2005 – 2010. Ministry of Health.

Although increasing numbers of women are becoming infected with HIV, they do not constitute a homogeneous group with similar levels of risk. Women involved in commercial sex work are known to be at higher risk of HIV infection [10,11], but the number of FSW infected with HIV may account for a small proportion of the total infected females, because they are so few among the population. The estimate was that by 2005, the number of HIV infected FSW would be 10,900, only 11% of the total estimated number of HIV positive women (98,500). Women not involved in commercial sex may account for a relatively high number of HIV infections even though their HIV prevalence is low, just because they are so many. The estimations also predicted that women in rural areas would account for 53% of the infections in women (Figure 2) [4].

In the following sections, the features of the epidemic in identifiable subgroups of women are described.

### Female sex workers

Within the sub-group of the commercial sex workers, there are identifiable sub-populations. FSW in Vietnam are often divided into two types for epidemiological estimations: street-based sex workers (SSW) and karaoke-based sex workers (KSW). The Ministry of Labor, Invalids and Social Affairs estimated that there were around 48,000 FSW in the whole country in 2004 [12]. SSW is classified as FSW who do not work in formal establishments, but on the street, in alleys or similar places. Their primary employment is selling sex. KSW work in entertainment establishments such as karaoke bars or massage parlors and provide other services depending on where they work. A new type of FSW is the call-girl, contacting clients by telephone or through websites; it is difficult to estimate their numbers and little is known about them. SSW differ from KSW in that they are older (mean age of SSW is 25–34, of KSW 21–24 years), have a lower educational level, have been working longer, have more clients, and are more likely to use drugs [13-16].

Research in Vietnam has revealed that a high proportion of FSW sell sex to get money to support an injecting habit [17-22]. They often live with male IDU; the men act as pimps to protect them in their work, share their income and share drugs with them [16,18,19]. Injecting FSW are therefore involved in double risk behavior, unsafe sex as well as unsafe injection. FSW should be classified as injecting or non-injecting because of their different levels of risk for HIV transmission. The proportions of drug users among FSW reported from different studies vary widely (Table 3), which may be a result of differences in the study methodologies or in the study populations in different ecological areas.

**Table 3:** Proportion of female sex workers injecting drugs

Province	Year	Population	Sample size	Proportion injecting	Source
Hanoi HCMC	2000	KSW	943	4.3% – 5.6% (last six months)	BSS [16]
Hanoi HCMC	2000	SSW	723	15.6% – 21.5% (last six months)	BSS [16]
Twelve provinces	2002	KSW	2,968	0.8% (ever)	[13, 14]
Twelve provinces	2002	SSW	1,185	9.1% (ever)	[13, 14]

On average, FSW reported having 2–18 clients per week [14,16,23,24]. There was a reported increase in consistent condom use with clients, rising from 33.3% in 1994 to 60% on average in 2000–2002 and to 84% in 2004 [14,15,23,25,26]. Despite the increased proportion of consistent condom use among clients, FSW also reported a tendency to use condoms less often with their regular partners, in particular with lovers with whom they share needles/syringes to inject drugs. The proportion of FSW having at least one sexually transmitted infection (*Chlamydia trachomatis*, *Gardnerella vaginalis*, *Trichomonas vaginalis*, gonorrhea, genital *Candida*, syphilis, genital warts) was more than 30%, reflecting the inconsistent use of condoms among this group [16,27].

Table 4 shows that the multiple risk behaviors of FSW resulted in a high prevalence of HIV in that population. Sentinel surveillance in 30 provinces revealed that HIV prevalence among FSW increased from 0.6% in 1994 to 4.2% in 2005 [1,28]. The provinces where HIV prevalence among FSW was higher than 14% in 2004 included the key urban centers of Hanoi, Cantho, Ho Chi Minh City, and the border zone of Angiang.

**Table 4:** HIV prevalence among female sex workers

Province	Year	Population	Sample size	HIV prevalence	Source
Ho Chi Minh city	2000	Non-DU FSW	259	12.4%	[56]
		Non-injecting DU FSW	58	27.6%	
		Injecting DU FSW	53	67.9%	
Ho Chi Minh City	2001	FSW	800	25%	[27]
Ho Chi Minh City	2002	FSW	306	26.8%	[27]
Twelve provinces	2002	FSW	4,153	4.7%	[13, 14]
Ho Chi Minh City	2003	FSW	381	11%*	[27]
		DU FSW	61	44.3%	
Hai Phong	2004	FSW	215	29.8%	[15]

* The lack of increase in seroprevalence and even the reduction in 2003 in HCMC is probably a result of the transfer of a high number of FSW to rehabilitation camps following the initiation of Three Reductions Program (reduction of drug use, prostitution, and crime) in Ho Chi Minh city in 2001.

### Women with risks through their partners

Women who are not engaged in direct high risk behavior may still be at risk because of the behavior of their male partners who may be IDU or clients of sex workers. In the following sections, we look different types of risk behavior among male partners, in decreasing order of risk. First we consider IDU partners, who also often have extramarital sex with commercial sex workers and other partners – a very high risk group. We then look at the risks from migrant workers, whose main risk may come from their interactions with commercial sex workers or other extramarital relations, but who also do use drugs and share needles, to some extent. The third type of risk behavior by the male partners is extramarital sex with commercial sex workers (who may also be female IDU). The last risk behavior described is pre-marital sex, which can involve multiple partners and may involve commercial sex workers.

#### Potential transmission from IDU partners

The countries of Laos, Myanmar (Burma), and Thailand, which make up what is known as the Golden Triangle, have become the most important source of heroin for Vietnam. This position has contributed to a growing epidemic of injecting drug use in Vietnam. The Ministry of Labor, Invalids and Social Affairs (MOLISA) estimated the number of heroin users at around 145,000 in 2004; of these, 70% were IDU [12]. Sentinel surveillance in 30 provinces showed that the HIV prevalence rate among IDU increased from 10% in 1996 to 32% in 2001 then remained stable until 2004.

The sexual habits of the male IDU will greatly influence the rate of transmission of HIV infections out of the IDU population. Studies revealed that 63–79% of IDU reported ever having had sexual intercourse [29,30]. In 1997–1999, around 30% of IDU reported they had had sex with a FSW in the past 12 months. One fifth reported having had anal sex [29,31]. Results from the first round of the Behavior Surveillance Survey (2000) showed a decline in this proportion as well as variation among different provinces 16. However, baseline surveys at hot spots with high HIV prevalence in 12 provinces (2002) indicated that 13–59% of IDU had had sex with a FSW in the previous 12 months. Additionally, studies in 1997–1999 showed that a high proportion of IDU reported never having used a condom with their sexual partners (37.5–62%), as well as a low rate of consistent condom use (less than 40%). Since 1997, although IDU tended to use condoms with FSW more consistently, that rate never rose above 60%. Potentially troubling is the fact that only 3–29% reported always using condoms with their lover or wife, and that IDU usually did not tell their sex partners about their drug use or HIV status [13,14]. Many women whose partners are IDU do not themselves use drugs, but they do not feel able to communicate with their partners about the risks that the partner's drug use and sexual habits impose on them [32]. The combination of unsafe drug use and unsafe sexual practices especially with wives or lovers would favor the transmission of HIV into the non-drug using female population.

#### Potential transmission from migrant population

People migrate from their place of living usually because of poverty and often move to legal or illegal sources of employment in the cities. According to an unofficial estimate, as many as 700,000 people move to urban areas every year, the majority of whom are men [33]. Male migrants may find work as long-distance truck drivers (LDTD), construction workers, workers in new economic zones, seafarers, traders, and taxi and motor scooter drivers. Studies in many countries have demonstrated that workers in these trades have contributed to spreading the HIV epidemic from high risk groups to the general female population [34,35].

The combination of having better incomes, being young and away from home means that migrants tend to engage in casual and/or commercial sexual relationships and in drug use. Two studies conducted in 2002 in 12 provinces that had increased HIV rates, highly mobile populations, and a border crossing with Cambodia or Laos revealed that up to one third of male migrants reported having had sex with FSW in the previous 12 months. Half of them were inconsistent in using condoms with FSW; less than one fourth consistently used condoms with their casual partners. The proportion of male migrants who reported always using condoms with their regular partners was even lower, at 8–9%. As many as 6–10% of LDTD reported ever having used heroin, while 1–3% of migrant workers had used it. Among those who reported using heroin, 10–50% injected. In the provinces Nghean and Thanhhoa, two thirds of the LDTD who injected drugs reported having shared needles/syringes in the previous month. The highest HIV prevalence rates were found among LDTD in Thanhhoa (4%), who also reported injecting drugs and sharing needles/syringes. In the other provinces, HIV prevalence rates among male migrants were lower than 1% [8,13].

Male migration therefore also seems to be a potential path for HIV transmission from high risk groups to their sexual partners. A prospective study in the National Obstetric Hospital between 2000 and 2004 showed that approximately 50% of the sexual partners of HIV positive pregnant women attending the hospital were working in the informal sector, either jobless or working as LDTD and migrant laborers [36].

#### Extra-marital sexual relationships

If FSW are one high-risk population, then men who visit FSW and do not use condoms are also at risk for infection. They then impose this risk on their wives (or other sexual partners) with whom they seldom use condoms, as discussed above. Up to now, there are no data from good population-based studies on the proportion of men who visit sex workers in Vietnam. A study in Hanoi in 2002 indicated that one-third of males aged 18–55 had ever had sex with a FSW; among that one-third, 45.3% had visited FSW more than five times in their life. Additionally, only 36.4% reported that they always used a condom with FSW [37]. This figure was higher among migrant workers, among whom one third reported having had sex with FSW within the preceding 12 months [13]. A cross-sectional survey in Quangninh revealed that 21% of male respondents had had multiple sexual partners and 13% had extra-marital sex [38].

No data are available on the frequency of extramarital sexual relations among women; in the absence of data, we can only note that anecdotal observation suggests that it is not infrequent and could occur often enough to create risks of HIV transmission for many women.

#### Pre-marital sexual relationships

Sex before marriage could also create risks especially if young men go to FSW who may be infected and then transmit infection to their future wives. Surveys among unmarried youth aged 14–25 in 42 provinces in 2003 showed that 15% of males and 2.5% of females had engaged in pre-marital sexual relations and that the average age at first sexual intercourse was 19.

*"Because of doi moi (opening of the economy), things are much more open, which often leads to excessiveness. For instance when in love, the woman can't know if the man truly loves her while she shows all her love for him. If you meet a 'So Khanh' (playboy) and you are easy, he will behave terribly after [having sex]." (Woman, Ha Ly*) [39]

Notably, 21.5% of single male youths reported having ever had sex with a FSW [40]. Three studies conducted in HCM city found that the proportion of unmarried students having had pre-marital sexual relationships increased from 5.5% in 1997 to 13% in 1998 and to 26% in 1999 [41,42]. The proportion of those reporting FSW as sexual partners showed a similar trend. A baseline survey among unmarried youth who had had pre-marital sex revealed that the proportion of young men who had had sex with FSW varied from 1.3% in Laichau to 35% in Longan. The proportion of youths who reported using a condom consistently with FSW also varied greatly, from 13.5% in Angiang to 83% in Laichau. The average use of condoms was 47%, similar to the proportion found among adult men reported above [13,43].

Household surveys in Ho Chi Minh City, Longan, and Laichau revealed a rather high HIV prevalence among youth aged 15–24: 1%, 2.6%, and 1.3%, respectively [6,13,43].

#### What did women do to protect themselves?

Women do know about HIV/AIDS. Results from many studies demonstrated that more than 90% of women aged 15–49 had ever heard about HIV/AIDS. Between 67% and 81% of women knew that a healthy-looking person can have HIV and 82.5% said that condom use could protect against HIV transmission. About 85% of women said that they would refuse sex or demand use of a condom if their sexual partner had a sexually transmitted infection [6,7,44].

In contrast, qualitative research revealed that in reality, the communication about sexuality between male and female partners was limited. Women often felt "ashamed" when they talked about sex and sexuality with their partners, it made them seem "unfaithful" or "amorous". Condom use was less among married couples since the condom was considered to show "an unfaithful man" or "losing emotion", although women thought that extra-marital sexual relationships were common among men [45-47]. A national survey among 10.6 million married women aged 15–49 revealed that only 8.4% of them used condoms for contraception [48]; married couples reported that they did not use condoms consistently through the whole month [44]. Even among discordant couples (HIV-positive male and HIV-negative female), only 60% reported sometimes using a condom [49]. Another study revealed that only 13% of women who had risk behavior also perceived that they could be at risk for HIV infection [6].

Reproductive tract infections, including sexually transmitted infections (STI), are very common among Vietnamese women, but estimates about the scope and severity of the problem vary. The prevalence of women having at least one reproductive tract infection has been estimated at 21 to 44% [50]. A survey conducted in Hanoi, Vinhphuc and Thaibinh found that 53% of 4,000 adolescents interviewed (aged 10–19) reported having symptoms of reproductive tract infections (RTI) [51]. In a survey among women aged 15–49 in four provinces, 16% reported having had an RTI within the past 12 months [7]. Moreover, among women in HCMC and Thaibinh aged 15–49 who had had an STI in the past 12 months, only 68% had sought treatment [6]. Women would be at greater risk of infection with HIV from their sexual partner if they had a reproductive tract infection (RTI) and did not seek or obtain proper treatment. As one woman put it:

*"Vietnamese women are used to suffering." (Woman, An Hung*) [32]

## Discussion

Women in Vietnam are increasingly at risk of HIV transmission. The HIV prevalence among women attending antenatal clinics has been rising annually. The first female case was found in 1990. Projections based on sentinel surveillance data using standard modeling software suggested that fifteen years later, the numbers of infected women would increase and the male-to-female ratio would decrease from 6 to 2.03. The number of women reported as HIV infected in 2005, however, accounted for only 16% of the estimated number and the male-to-female ratio had remained stable, not decreased. This could be interpreted in two ways: either the predictions were wrong, or there is serious under-reporting for a variety of possible reasons. Because the predictions were made using relatively reliable sentinel surveillance data and standard UNAIDS software and methods, we expect that the problem lies in the detection and reporting of HIV positive women. Although there is undoubtedly under-reporting for men as well, the difference between the predictions and the reported numbers is less; reported cases in 2005 accounted for 40% of the expected number of male cases. Unless changes are made in the ways that women can be reached by the health system, the risk is great that the vast majority of HIV-positive women will go without appropriate care and treatment. The main issue is that when most of the programs focus on the predominant risk group (male IDU), they increase the risk that others (especially women) go unaware of their risks, undetected and therefore without the care they need.

Especially because many of the HIV-infected women form the first line of transmission of the virus into the general population, it is important that programs for prevention and care are able to detect risk groups among women. To make that possible, it is necessary to find out which women are at risk and how many they are, whether the estimates were too high or the detection rate is too low.

**Figure 3:**
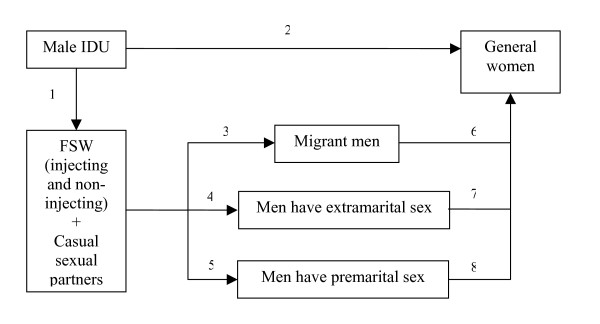
Possible routes for transmission of HIV to women in Vietnam.

Potentially, different routes could lead to infections among women. Nearly all of them would come through male partners, who either used drugs, or had sex with infected FSW or with infected men (Figure 3) [52,53]. The number of women who might become infected through blood products or occupational exposure should be minimal and is not shown. Most new infections among women can be traced to sexual relations with young male injecting drug users engaged in extramarital sex (in the diagram, arrows 1 and 2) [29].

Transmission through a non-IDU man who has visited a sex worker is possible (arrows 6, 7, 8) although the data for this group are fewer and more difficult to collect or verify. Very few data are available on men who have sex with men but also with a female partner. Sex with FSW is more common and more open these days and in the more industrialized areas [13,14].

Female sex workers who may also inject drugs could play an important role in HIV transmission to their male partners (arrows 3, 4, and 5 in the figure) in the general population in Vietnam. FSW who are addicted also tend to become more active in selling sex to cover the high cost of drugs, tend to use condoms less than those who are not addicted, and are therefore exposed to a higher risk of HIV transmission. However, data among this group is very limited. The 2003 estimations predicted that 17% of FSW would be infected with HIV in 2005 [4]. However, with their small population size and stable HIV prevalence, the HIV-infected FSW are expected to contribute only a small proportion to the 98,500 HIV-infected women in that year, as estimated by the Ministry of Health.

In this figure, the women who acquire their risk of HIV infection through their partner are invisible until they are detected because of an interaction with the HIV program, for example if they have a child who becomes ill and is detected as HIV positive. The casual sexual partners of IDU (and then of other men in risk groups) are also undetectable, while FSW, male IDU and to a certain extent the three male risk groups (arrows 6,7 and 8) can be reached by interventions aimed at prevention.

Without consideration of the risks of transmission along the routes shown in Figure 3, programmers and policy makers may make broad assumptions about appropriate target populations for their interventions. Most interventions are now focused on the visible high risk groups, while in reality the HIV epidemic has also been increasing among groups that might be expected to have low risk [2]. The data reviewed in this paper suggest that transmission to women who are not participating in high risk behavior may be increasing. They include the sexual partners of male drug users, at highest risk of HIV infection because of their unsafe injecting and unsafe sexual behaviors. But women may be at risk as sexual partners (pre-, intra-, and extra-marital) of men who have multiple sexual partners with whom they usually do not use condoms.

At this time in the epidemic in Vietnam, people in the general population are not yet clearly aware of the risk to them. Intervention strategies should be developed in time to reduce the transmission to all vulnerable groups and to detect and provide care for those already infected. Behavior change communication is now starting to be aimed at the general population but still most women would not consider themselves at risk; they need to receive better and more targeted information. Probably the most effective preventive approach would be to increase condom use especially among men who have sex with FSW. However, men who are willing to use condoms with women they see as FSW still prefer not to use them with women they see as more long-term partners, even outside their marriage. Also, condoms have been promoted in Vietnam mainly as contraceptives, with relatively little success; the burden of contraception is still on the women [54]. The routes for distribution and counseling on condoms are mainly through family planning centers. Condoms should be made widely available to the general population, with priority to youth and aimed at dual protection, that is, at prevention of both unwanted pregnancy and of STI including HIV.

Another need is to increase the detection of HIV-infected women. Voluntary counseling and testing (VCT) targeting non-high risk populations and routine testing in antenatal care could help to bring the numbers of HIV-positive women detected closer to the expected real number. However, VCT for low-risk groups is not cost-effective. Also, at present only a few antenatal care centers and maternity hospitals offer VCT routinely and reliably. The majority of these are at provincial level in urban areas, while as many as 70% of pregnant women use ANC at district or lower level facilities, where testing is not yet widely available [54]. Therefore, just improving the existing HIV testing service could not cover the targeted population in rural areas, estimated to account for 53% of total female infections. Not even all the women in urban areas are covered, as HIV testing is not yet widely available even in well-resourced settings. Even if the service is available, the stigma attached to HIV infection, drugs and sex work, and the lack of confidentiality in the present notification system may limit accessibility and acceptability of HIV testing services [55]. A different approach is needed to increase the HIV detection rate, offering confidential and accessible HIV tests and taking into account the factors of culture and cost-effectiveness.

## Conclusion

The HIV epidemic in Vietnam has been perceived as predominantly male IDU and the number of women infected with HIV seems to be strongly under-reported. There is an observation of increasing HIV prevalence among the general population and an increasing risk of HIV transmission among women who do not use drugs or engage in sex work. They presently receive less attention from HIV intervention programs in Vietnam. More effort should be given to more effective interventions, making information, testing and care more widely available and accessible to this population.

## Competing interests

The author(s) declare that they have no competing interests.

## Authors' contributions

Concept protocol: TAN, AH, PW, HNT, RC

Data collection: TAN, HNT, PW, PO

Data analysis: TAN, PW, AH

Manuscript draft: TAN, PO, AH, PW, HNT, RC

## Pre-publication history

The pre-publication history for this paper can be accessed here:


